# *Corona Mortis*: A Systematic Review of Literature

**DOI:** 10.6061/clinics/2021/e2182

**Published:** 2021-04-07

**Authors:** Giovana Irikura Cardoso, Lucas Albuquerque Chinelatto, Flavio Hojaij, Flávia Emi Akamatsu, Alfredo Luiz Jacomo

**Affiliations:** I Faculdade de Medicina de Marília (FAMEMA), Marilia, SP, BR; II Faculdade de Medicina FMUSP, Universidade de Sao Paulo, Sao Paulo, SP, BR; III Departamento de Cirurgia, Faculdade de Medicina FMUSP, Universidade de Sao Paulo, Sao Paulo, SP, BR

**Keywords:** Corona Mortis, Surgery, Anatomical, Variation, Vascularization, Pelvis

## Abstract

Our systematic review evaluates surgically relevant information about *corona mortis (CM),* such as anatomical structure, size, laterality, incidence, and anthropometric correlations. This study aimed to provide data about anastomosis in an attempt to avoid iatrogenic damage during surgery. Articles were searched online using the descriptor “*Corona Mortis*” in PubMed, Biblioteca Virtual em Saúde (BVS) (Literatura Latino-Americana e do Caribe em Saúde [LILACS], MEDLINE, indice bibliografico espaãol en ciencias de la salud [IBECS]), and SciELO database. The time range was set between 1995 and 2020. The articles were selected according to their titles and later the abstracts' relation to our research purpose. All the selected articles were read entirely. A manual search based of the references cited in these articles was also conducted to identify other articles or books of interest. Forty references fulfilled the criteria for this review. The mean incidence of *CM* was 63% (the majority venous) among 3,107 hemipelvises. The incidence of bilateral *CM* was lower than that of unilateral variations based on the analysis of 831 pelvises. The mean caliber of the anastomosis was 2.8 mm among 1,608 hemipelvises. There is no consensus concerning the anthropometric influences in *CM*. Finally, we concluded that *CM* is not an unusual anatomical variation and that we must not underestimate the risk of encountering the anastomosis during surgery. Anatomical knowledge of *CM* is, therefore, essential in preventing accidents for surgeons who approach the inguinal and retropubic regions.

## INTRODUCTION

The blood vessels related to the pelvis and the abdominal wall are susceptible to anatomical variations, especially the inferior epigastric and obturator vessels, which cross the superior pubic ramus and the lacunar ligament in the inguinal region. The inferior epigastric vessel usually branches from the external iliac system, and the obturator vessels are usually branching of the internal iliac systems (1). The connection of these two main systems by anatomical vessel variation is known as *corona mortis (CM)* (2).

*Corona Mortis* stands for “Crown of Death” in Latin because a lesion in this structure may result in significant bleeding (3). The most common iatrogenic damage occurs in surgical and orthopedic interventions, such as inguinal or femoral hernia repairs and hip fractures (4). This vessels’ classification is discussed in the literature, where articles classify its anatomical structure as aberrant, anomalous, or accessory (5,6).

*CM* can be arterial and/or venous and can also be classified as bilateral or unilateral. Most studies have focused on the arterial anatomical variation because lesions associated with it are more severe than the venous one. The lesion of the venous *CM* is more difficult to identify (7).

The topic has already been addressed in different types of studies such as bibliographic reviews (8,9), case reports, cadaver dissections, studies based on image examination (10-12), and surgeries (13,14). There is a lack of consensus regarding some aspects of *CM*, resulting from contradictory data among different studies. Simultaneously, clear comprehension of the *CM* is needed, as this structure presents a high risk for lesions during surgery (3).

The purpose of this systematic review was to assemble and analyze surgically relevant information about this anastomosis, such as the anatomical structure type and incidence of *CM*, caliber, laterality, and influences from anthropometric factors. Our aim is to provide a better comprehension of the anastomosis characteristics, in order make surgeons more aware about its management.

## METHODS

We conducted a literature review based on an online article search. The following platforms were used as databases: PubMed, Portal Regional da Biblioteca Virtual em Saúde (BVS), which includes Literatura Latino-Americana e do Caribe em Ciências da Saúde (LILACS), Medical Literature Analysis and Retrieval System Online (MEDLINE) and ĺndice Bibliografico Espaãol en Ciencias de la Salud (IBECS), and Scientific Electronic Library Online (Scielo). The standard descriptor was “*Corona Mortis,”* and the analyzed time range was 25 years (1995-2020) because video-herniorrhaphy started 25 years ago. After excluding duplicates and articles that did not have an online version, the papers were included first according to their title and later based on their abstract's relation with the subject of interest. All articles with titles and abstracts that concerned the theme *CM* were read entirely. Moreover, a manual search based on the references cited in these articles was performed, and 13 new articles and books of interest were added to the review. To be included in this review, the reference had to contain at least one of the following characteristics: definition of *CM*, measurements of the caliber of the vessels, incidences of *CM* in each type of blood vessel, and their origins (obturator artery [ies]), and anthropometric influences.

The analysis of each article included in this review consisted of six steps: identifying the year of publication (checking if it was within the given 25-year range); identifying the type of article; identifying the *CM* definition in the article; identifying the morphometric measurements of vessel caliber, incidences, and whether there was information that could be applicable for this review i.e., for data analysis and comparisons with other papers to help organize the obtained data. Finally, data analysis and a combination of the studies' results were performed using Excel (Microsoft, Redmond 265 WA, USA). The means calculated were rounded to one or two decimals according to the data precision.

## RESULTS

**Figure 1:**
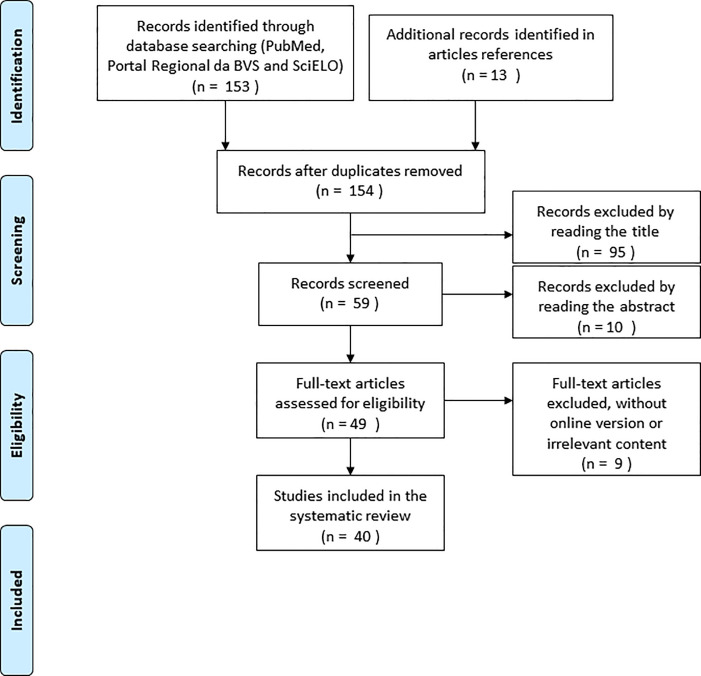
Inclusion Flowchart. BVS-Portal Regional da Biblioteca Virtual em Saúde, including LILACS (Literatura Latino-Americana e do Caribe em Saúde), MEDLINE (Medical Literature Analysis and Retrieval System Online) and IBECS (indice bibliografico espaãol en ciencias de la salud). SciELO- Scientific Eletronic Library Online.

A total of 153 articles were found in the search engines used (79 PubMed, 69 BVS, and 5 SciELO) and added to the 13 articles identified manually. Out of those, 154 remained after the removal of duplicates. Following the selection process, 40 articles were included in this review (Figure 1).

**Figure 2:**
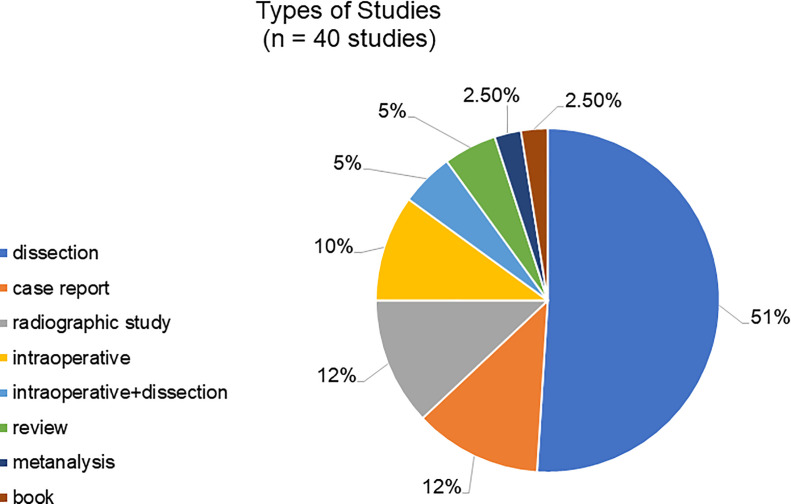
Types of Studies. n=number of studies considered in this graphic; dissection=cadaveric dissection; Intraoperative+dissection=studies that include surgeries reports and cadaveric dissections. Vascular radiographic studies are mainly pelvic or lower extremity angiographies. Intraoperative studies are performed in hernia or acetabulum fracture repair surgeries, for example.

Regarding the type of study, among the 40 references, most were cadaveric dissections, followed by intraoperative studies, case reports, and others (Figure 2). The complete list of dissections and surgical or angiographic studies can be seen in Table 1, which also presents the number of hemipelvises in each study.

### Incidence of *CM*

**Figure 3:**
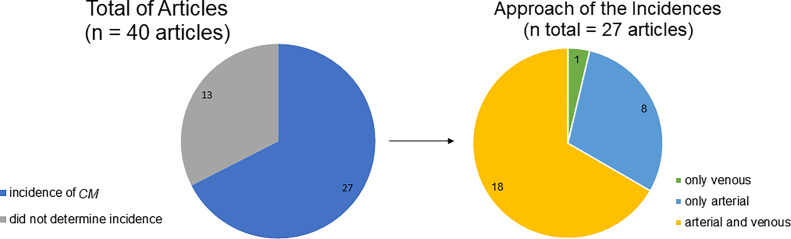
Incidence of *Corona Mortis*. *CM: Corona Mortis*. n: total number of articles considered in each graphic.

The incidence of *CM* was analyzed in 27 articles. There were different methodologies: 18 provided data on venous and arterial *CM*, eight provided data on arterial *CM* only, and one addressed venous *CM* only (Figure 3).

**Figure 4:**
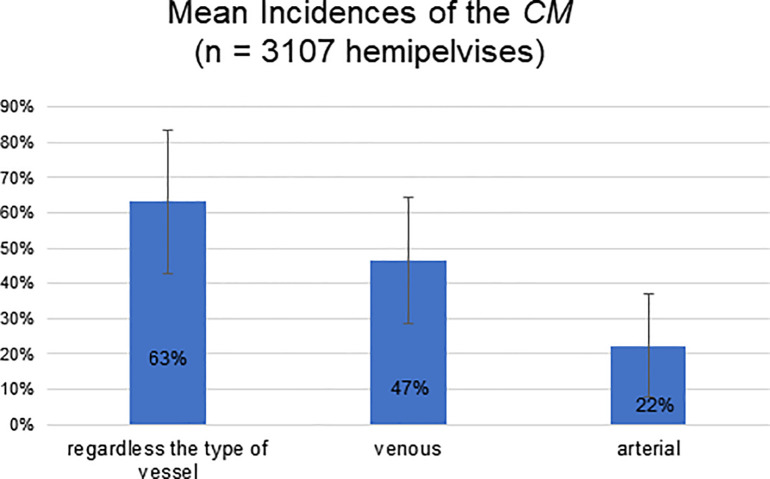
Mean Incidences of *Corona Mortis.* n=total number of hemipelvises considered in this graphic. The standard deviations are indicated as bars in the figure, and the percentages are calculated considering hemipelvises. In studies performed with cadavers and surgical patients, data was compiled to a single analysis, except in one of the studies (33), in which only the cadaveric research is used because, in surgeries, it does not accurately discriminate arteries from veins.

From the 18 studies approaching venous and arterial *CM* (3,107 hemipelvises), it was calculated that the mean incidence of *CM*, regardless of blood vessel type, was 63%±20% (maximum 96% and minimum 20%). Based on the analysis of the 26 articles that approached arterial *CM*, the calculated mean incidence of arterial *CM* was 22%±14% (maximum 68% and minimum 0%). Finally, the analysis of 19 articles approaching venous CM presented a mean incidence of 47%±18% (maximum 88% and minimum 17%). The data summary is shown in Figure 4.

### Anastomosis caliber

The anastomosis caliber was analyzed in 11 articles (1,608 hemipelvises), which are presented in Table 2. The maximum diameter of the *CM* was 4.9 mm, while the minimum was 0.8 mm, and the mean diameter was 2.8 mm.

### Laterality

**Figure 5:**
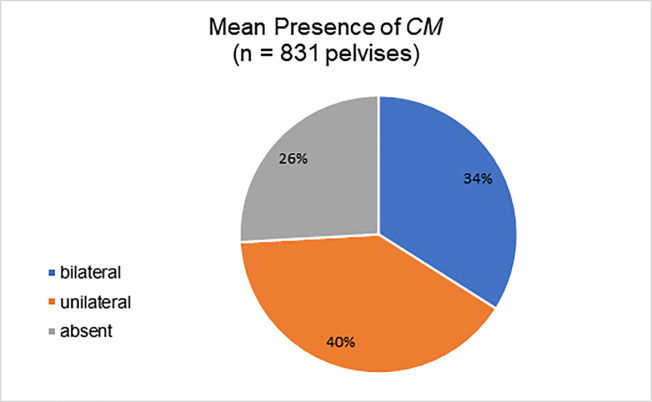
Presence of *Corona Mortis*. *CM=Corona Mortis*; n=total number of pelvises analyzed (7,11,12,18,20,21,27,31,33,39).

Among the evaluated studies, 10 articles (a total of 831 pelvises) considered the laterality of *CM* (Figure 5). There was no significant difference in the occurrence of unilateral *CM* on the right side compared to the left side (27).

### Anthropometric influences

Ten articles evaluated possible ethnic correlations with the incidence of *CM*. Most articles presented findings related to genetic variations with vascular variations, including *CM*. Studies that have evaluated the genetic influence on *CM* have contradicted each other. Additionally, when ethnicity is taken into consideration in the articles, there is only a qualitative analysis.

The sex differences were addressed in only four articles. There were no significant conclusions on this topic. A greater number of studies in male participants was observed, as presented in Table 1.

## DISCUSSION

We conclude that *CM* is occurs more frequently than it is reported in the literature (1). The high incidence of this anastomosis in practical studies (present in 63% of hemipelvises) contradicts the widely disseminated feature of *CM* as an anomalous or aberrant structure. The presence of these blood vessels is a common alteration that, although it is often challenging in surgery, constitutes an important network of collateral vascularization of the pelvis and abdomen in aortoiliac and femoral arterial occlusive diseases, which was not the focus of this study (4,22).

Based on our findings, the incidence of *CM* in cadaveric studies was higher than that in intraoperative articles. Postmortem conditions, fewer intraoperative studies with cadaveric dissections, and blood-vessel “spasm” in pelvic fractures (16,21,40) may explain this difference in incidence. We believe that intraoperative anatomical studies are susceptible to criticism. Moreover, there is a great lack of standardization of *CM* definition and terminology, which hampers anatomical analysis.

Although there is a predominance of venous *CM* compared to arterial *CM*, except in one study (23), we consider that anastomosis is important in both types of vessels. Indeed, the *CM* represents a connection between the two major vascular systems (external and internal iliac); therefore, any injury is potentially serious. Hence, surgeons should be aware of the presence of *CM* during surgery. In this regard, our findings suggest that *CM* bilaterality is less common than unilaterality. Thus, if the *CM* is found in one hemipelvis in bilateral pelvic surgeries, it is less likely to be found in the other.

We did not reach conclusions about any anthropometric influences in *CM* in our review. This was mainly due to inconsistencies in data among the analyzed studies. Further studies should be encouraged to establish relations that could alert surgeons regarding the presence of *CM* before the procedures.

Finally, it should be emphasized that some precautions can be taken to avoid *CM* injury, such as angiographic mapping of the pelvis before surgery. Even so, iatrogenic damage and consequent *CM* injury are possible, and in this case, there are means to repair the lesion efficiently other than converting to open surgery (14). A successful hemostasis technique by embolization of blood vessels has been reported by some authors (18,37). Still, caution must be taken to avoid hemorrhage and any injury to the obturator nerve along the way.

This study has some limitations, such as the inability to review some articles that were in our time range but not fully accessible. Furthermore, the included articles diverged in methodology, making a meta-analysis unfeasible, allowing only a gross calculation of incidence. Moreover, this review did not evaluate the origins and divisions of the obturator artery, though some studies (40) do, because our review focused only on *CM* incidence. In this study, ethnically different populations were analyzed; thus, a faithful portrait of the Brazilian population may not have been illustrated. Finally, the study bias was not assessed.

## CONCLUSION

Our main finding is that the mean incidence of *CM* is 63%, which suggests that it is not as rare as it is believed to. The *CM* mean caliber was calculated to be 2.8 mm, with a more unilateral incidence and no specific side incidence. We found no anthropometric influence on the *CM* characteristics.

Surgeons certainly must not underestimate the risks of coming across the anastomosis during surgeries, as our studies suggest that the *CM* is present in more than half of the population. There is a need for further research on *CM*, including in Brazil, to estimate better the incidence of *CM* in the Brazilian population.

## AUTHOR CONTRIBUTIONS

Cardoso GI and Chinelatto LA were responsible for the data acquisition, analysis and interpretation, statistics analysis, manuscript preparation and writing. Hojaij F was responsible for the manuscript preparation and writing. Akamatsu FE and Jacomo AL were responsible for the study supervision.

## Figures and Tables

**Table 1:** List of Dissections and Surgical or Angiographic Studies.

Reference	Number of hemipelvises/cadavers studied	Type of study
(15)	73 hemipelvises (4 female and 69 male)	Cadaveric dissection
(16)	10 hemipelvises (4 female and 6 male)	
(17)	60 hemipelvises	
(18)	30 female hemipelvises	
(2)	40 hemipelvises	
(4)	60 hemipelvises (10 female and 50 male)	
(19)	28 hemipelvises	
(20)	150 hemipelvises (34 female and 116 male)	
(21)	24 hemipelvises (2 female and 22 male)	
(22)	98 hemipelvises (36 female and 62 male)	
(23)	14 hemipelvises (16 female and 24 male)	
(24)	50 hemipelvises	
(6)	50 hemipelvises (6 female and 44 male)	
(25)	80 hemipelvises (26 female and 54 male)	
(26)	54 hemipelvises (4 female and 50 male)	
(27)	98 hemipelvises	
(7)	70 female hemipelvises	
(28)	208 hemipelvises (56 female and 152 male)	
(5)	105 hemipelvises (45 Americans and 50 Chinese)	
(29)	24 hemipelvises (8 female and 16 male)	
(30)	96 hemipelvises	Intraoperative (lymphadenectomy)
(31)	50 hemipelvises	
(13)	141 hemipelvises	Intraoperative (hernioplasty)
(14)	398 hemipelvises	
(32)	14 hemipelvises 36 hemipelvises	Cadaveric dissection Intraoperative (laparoscopy)
(33)	79 hemipelvises 38 hemipelvises (8 female and 30 male)	Cadaveric dissection Intraoperative (acetabular fracture repair)
(34)	1 male cadaver	Case report (cadaver)
(35)	1 male cadaver	
(3)	1 female cadaver	
(36)	1 male cadaver	
(37)	1 man	Case report (intraoperative)
(10)	96 right hemipelvises (39 female and 59 male)	Vascular radiographic study
(38)	98 female hemipelvises	
(11)	300 hemipelvises (76 female and 224 male)	
(12)	200 hemipelvises (68 female and 132 male)	
(39)	660 female hemipelvises	

**Table 2:** Caliber of the Anastomosis.

Reference	Caliber of the *CM* (mm)	Hemipelvises
(4)	2.66±0.5	60
(19)	2	28
(10)	2.0-4.0	96
(23)	2.0-4.2	14
(24)	2.0-4.0	50
(25)	1.6-3.5 (average=2.6)	80
(32)	2.2-4.9 (average=3.3)	50
(7)	3.0-3.13	70
(11)	0.8-3.2 (average=1.7)	300
(12)	1.4-3.7 (average=3.32)	200
(39)	a:2.56±0.73 v: 3.67±0.84	660
**Estimated average**	**2.8**	**1608**

*CM=Corona Mortis*. Not all articles distinguish the caliber from venous to arterial *CM*; those distinguishing with relevant differences are identified in the table by a (arterial) and v (venous).
